# Analysis of Fractional-Order Models Of Polyaniline Doped Polyacrylonitrile Fibres Impedances’ (PAN/PANI)

**DOI:** 10.1038/s41598-020-57746-9

**Published:** 2020-01-21

**Authors:** Tomasz Rybicki, Iwona Karbownik

**Affiliations:** Lodz University of Technology, Institute of Automatic Control, Lodz, Poland

**Keywords:** Characterization and analytical techniques, Characterization and analytical techniques, Computational methods, Scientific data, Scientific data

## Abstract

The paper describes the use of electrical impedance spectroscopy (EIS) for characterizing the impedance of polyaniline doped polyacrylonitrile fibres. The electrical impedance of fibres samples was measured by means of high impedance analyser and modeled by four types of models: Debye, Cole-Cole, Davidson-Cole and Havriliak-Negami. The fitting errors are presented for all model types. The model parameters are correlated with chemical substances additives and processing which were used during the fibres production. The presented results show that the fractional-order models reflect properly the impedance spectra of fibres samples.

## Introduction

Electrical impedance spectroscopy (EIS) is widely used for measuring the electrical impedance of tested materials over a given range of frequencies^1^. It returns the complex impedance spectra in the frequency domain. EIS is used for characterising different types of substances and materials. Electrical impedance spectroscopy results play an important role in the modelling process of new and designed materials. Modelling the behaviour and response of materials is important for various reasons. Namely, prediction of standard and anomalous phenomena is possible by the use of model analysis and model-based simulation. Moreover, in many applications, the models allow to evaluate performance indexes and to adjust the structure or material’s parameters so that responses obey design specifications and reference behaviours^2^.

Textile materials (various clothing and technical fabrics) are made of polymeric, natural or chemical fibers, in particular synthetic ones. These fibers are mainly polymers with poor electroconductive properties (e.g., polyacrylonitrile fibers are dielectrics with a resistance of 10^11^ Ω). Among textile materials there is a group of smart/intelligent textiles that are sensitive to external stimuli such as stress, temperature, humidity, the intensity of the electromagnetic field or the presence of chemical substances. Under the influence of the above-mentioned external factors, the textile material changes its properties, e.g. volume, color or electrical conductivity^3,4^. The electrically conductive polymer fibers are of great importance^5^. Electroconductive fibers, depending on their resistance, can be used as electrical connections, sensors or electromagnetic field shielding materials. However, the mechanical properties of the electrically conductive fibers must allow their further processing to obtain a textile product.

In this work, polyacrylonitrile-polyaniline fibers (PAN/PANI) were made. The polymer matrix for these fibers was polyacrylonitrile and admixture - an electrically conductive polymer - polyaniline. Polyaniline is an intensely tested material mainly due to its high electrical conductivity, low price and an easy method of obtaining. However, the biggest difficulty associated with its use is its low mechanical strength and processing problems. For this reason, the fibers were made from a mixture of polyaniline with fiber-forming polyacrylonitrile, which in this system plays a role of polymer responsible for mechanical strength.

The impedance tests of the obtained fibers were to show the electrical static and dynamic properties of the obtained composite fibrous materials. An attempt was also made to explain the phenomena occurring in real systems based on model considerations. Traditionally, models of physical phenomena or different technical properties (electrical, chemical, mechanical, etc.) are created on the basis of differential equations of integer orders. Recently, however, more and more modeling attempts have emerged as a result of fractional order differential equations – fractional order models. They can be applied to describe different phenomena, for instance dielectric fractional models based upon relaxation phenomena^6^. It is related to the intensively developing discipline of science which is called fractional calculus^7^. Fractional calculus is a generalization of the integer order calculus known from basic education. By introducing one operator of arbitrary differentiation order - Davis operator, it allows, depending on the exponent sign, to express differentiation or integration of any order^8^:

1 $$a{D}_{b}^{n}f(t)=\frac{{d}^{n}f(t)}{d{t}^{n}}$$

where:

*n* – noninteger differentiation order

*a, b* – terminals of fractional differentiation.

In addition to the operator, one also needs definitions for any order derivative. There are several definitions of such kind, which differ in the accepted integration limits, the class of functions for which they apply and the range of values of the noninteger exponent *n* for which they remain valid: e.g. Grunwald-Letnikow definition, Riemann-Lioville definition or Cauchy definition^8,9^.

## Theory (Mathematical Background)

### Integer order models

As it was already mentioned in the Introduction section, the most popular models of technical phenomena are still integer order ones. As a basis, they use mathematical descriptions of physical phenomena expressed in the form of ordinary differential equations. They also provide a consistent interpretation of physical phenomena. In automatic control theory^10^, for example, one of the simplest models that can sufficiently reflect the dynamical behaviour of different dynamical objects and phenomena is first order system with the time constant. It is also called inertial model of first order. The object behavior according to the first order inertial model can be described by the following integer order differential equation:

2 $$T\frac{dy(t)}{dt}+y(t)=Ku(t)$$

where:

*u(t), y(t), K, T* – are respectively: input signal, output signal, gain constant and time constant.

Performing a Laplace transform at zero initial conditions and grouping the variables appropriately, one can get the transfer function of the first order inertial element. The transfer function, as the relationship between its output and input signals, using Laplace transform, can be represented by the Eq. (3):

3 $$G(s)=\frac{y(s)}{u(s)}=\frac{K}{1+sT}$$

where:

*u*(*s*), *y*(*s*) – Laplace transform of input and output signals respectively; *s* – Laplace variable, *s* = *jω* when frequency domain is considered.

On the other hand, in physics and electrochemistry, nearly the same relationship as (3) was acquired using experimental methods. It was done by Debye^11,12^, who was analysing the complex permeability of a homogeneous dielectrics and proved the existence of constant relaxation time that described the process of polarization decay after removing the external electrical field^13,14^. The process was described by a famous dispersion equation in relation to complex susceptibility (4)^15^:

4 $$\chi ={\chi }_{\infty }+\frac{{\chi }_{s}-{\chi }_{\infty }}{1+j\omega \tau }$$

where:

$\chi$– electrical susceptibility of the dielectric, ${\chi }_{\infty }$- induced susceptibility, $\tau$- relaxation time.

Comparing Eqs. (3) and (4) one can see that (3) is much more general because it deals with input and output signals of different type. Also the constant values in (3) – gain and time constant, can represent different phenomena where in (4) they are connected only with dielectric properties. Taking into account that ${\chi }_{\infty }=0$, ${\chi }_{s}$=*K* and $\tau =T$ in Eqs. (3) and (4) one can see they lead to the same relationship in frequency domain.

Comparing the formulas for the transfer function of the Debye dispersion Eq. (4) and the first order inertial element (3), it can be shown that the Debye equation is also derived from a integer order differential equation similar to (2).

In one of the simplest cases the impedance of real dielectric with losses can be represented in frequency domain by parallel connection of simple capacitive and resistive elements – Eq. (5):

5 $$Z(j\omega )=\frac{u(j\omega )}{i(j\omega )}=\frac{R}{1+j\omega RC}$$

where:

*u*(*jω*)*, i*(*jω*) *–* voltage and current signals respectively*; R*, *C* – dielectric resistance and capacitance respectively; *RC* – dielectric relaxation time constant.

Comparing Eqs. (5), (3) and (4) one can see that (5) represents the form of first order inertial element or Debye equation.

### Fractional-order models

The Debye’s dispersion equation (DEB) holds when simple relaxation model with single relaxation time is considered, neglecting the intermolecular interactions^15^. Shortly after the introduction of Debye model (4), it turned out that there still exist lots of different types of real dielectrics which process data of polarization decay do not fit that model. As a result, several different types of so called non-Debye models were introduced. The basic rule that led to non-Debye models was that they were generally based upon Debye model with small modifications. The simplest modification that led to better fitting of experimental data was the introduction of fractional exponent in (4). The model was introduced by two researches (Cole brothers) and called based upon their names Cole-Cole model (CC)^16–19^:

6 $$\chi ={\chi }_{\infty }+\frac{{\chi }_{s}-{\chi }_{\infty }}{1+{(j\omega \tau )}^{1-h}}$$

where:

*h* – fractional exponent, 0 < *h* < 1, h increases with increasing internal degrees of freedom of molecules and decreases as the temperature increases. The Eq. (6) becomes Debye’s equation for *h* = 0.

Using the transfer function form the Eq. (6) can be expressed as:

7 $${G}_{CC}(s)=\frac{y(s)}{u(s)}=\frac{K}{1+{(sT)}^{\alpha }}$$

where:

α – fractional exponent, $$\alpha \in R$$

In automatic control theory (7) is called inertial model of fractional order^9^. For $\,{\chi }_{\infty }=0$ and $\alpha =1-h$ Eqs. (6) and (7) lead to the same expression.

To prove that models (6) and (7) are actually fractional order ones, it must be demonstrated that they are based on fractional order differential equations. Replacing in the Eq. (2) the first order derivative operation with a fractional derivative using Davis notation, the differential equation takes the form:

8 $${T}_{0}^{\alpha }{D}_{t}^{\alpha }y(t)+y(t)=Ku(t)$$

Performing a Laplace transform at zero initial conditions at (8) and grouping the variables appropriately, leads to the transfer function (7).

The basic element of fractional order, both in electrochemistry and automatic control theory is the so-called fractional order integrator. In electrochemistry, it is the result of empirical considerations and is called the constant phase element (CPE). Its transfer function is presented in Eq. (9):

9 $${G}_{CPE}=\frac{1}{{(Ts)}^{n}}$$

and its fractional differential equation is presented in (10):

10 $$y(t)={T}_{0}^{-n}{D}_{t}^{(-n)}u(t)$$

The CPE element plays an important role in modelling complex electrochemical processes like anomalous diffusion proving the existence of complex relaxation phenomena. Much more complicated models are based upon the CPE model^20–22^. It can be proven that there are several reasons leading to CPEs^13^:

- porosity of the electrodes or the sample itself,
- diffusion phenomena involving reaction products near the electrode,
- local distribution of easy conduction paths,
- Debye-Huckl relaxation accompanying ion transport,
- statistical distribution of grain resistance in a ceramic sample.

**Figure 1:**
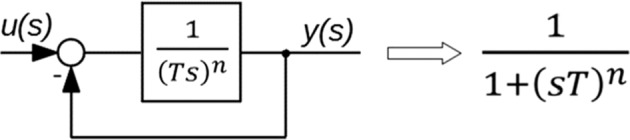
Closed loop system with CPE.

From the other hand in automatic control theory closed loop system with CPE (9) leads to inertial model of fractional order that is the general form of Cole-Cole model (7)^9^. The process can be schematically shown in Fig. 1.

The schematic diagram shown in Fig. 1 proofs the clear connection between CPE and Cole-Cole model. The Cole-Cole model is the result of internal feedback that occurs on the way from input to output signal especially when the CPE is involved.

For different and more complicated classes of objects (for instance: chemical substances like glycerol and n-octyliodine^15^) another modification of Debye model was introduced that led to Davidson-Cole approach (DC)^23,24^:

11 $$\chi ={\chi }_{\infty }+\frac{{\chi }_{s}-{\chi }_{\infty }}{{(1+j\omega \tau )}^{l}}$$

where:

*l* – fractional exponent, 0 < l < 1

For *l* = 1 we obtain Debye’s equation.

In transfer function form, the Davidson-Cole model can be described as:

12 $${G}_{DC}(s)=\frac{K}{{(1+sT)}^{\gamma }}$$

where:

γ – fractional exponent,For gamma = 1 it expresses the (6) equation.

The most advanced form of the non-Debye model can be composed based upon (4) and (6) that leads to Havariliak-Negami model^25,26^:

13 $$\chi ={\chi }_{\infty }+\frac{{\chi }_{s}-{\chi }_{\infty }}{{(1+{(j\omega \tau )}^{1-h})}^{l}}$$

The transfer function form of equation can be represented as follows:

14 $${G}_{HN}(s)=\frac{K}{{(1+{(sT)}^{\alpha })}^{\gamma }}$$

where:

α, γ – fractional exponent, $$\alpha ,\,\gamma \in R$$

It can be also proved that both Davidson-Cole and Havariliak-Negami models can be also derived from fractional differential equations but the mathematical expressions are much more complicated compared with Debye and Cole-Cole cases.^27^.

**Figure 2:**
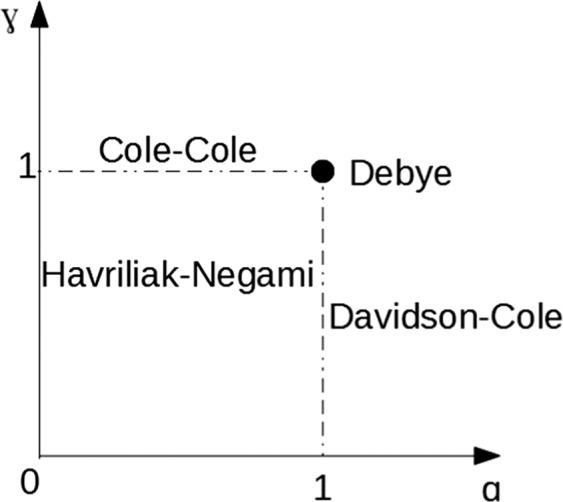
Locus of Debye, Cole-Cole, Davidson-Cole and Havriliak-Negami models.

The locus, in respect to fractional exponents, of Debye, Cole-Cole, Davidson-Cole and Havriliak-Negami is presented in Fig. 2^28^.

Figure 2 shows the presented models on α, γ parameters plane. It can be seen that Debye model with fixed α = 1, γ = 1 parameters is represented by one point. Cole-Cole and Davidson-Cole models have one degree of freedom and are represented by segments. Only the Havriliak-Negami model covers the whole plane as a result of two degrees of freedom.

**Figure 3:**
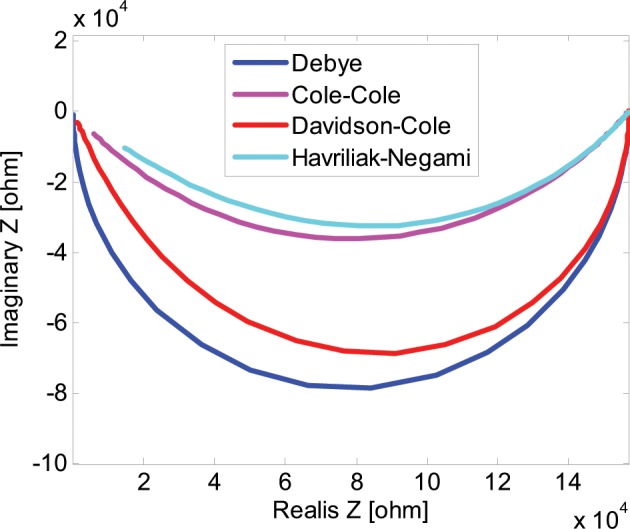
Nyquist (inverted Cole-Cole) plots of Debye and non-Debye models. The following values of the parameters were used in the models: K = 1.57e5, T = 2.9e-5, α = 0.55, γ = 0.75. The frequency range considered was from 0.01 Hz to 100 kHz.

Figure 3 presents some examples of the Nyquist plots of models presented by Eqs. (3, 4), (6, 7), (11–14) when changing the frequencies from ω = 0 to ω = ∞. The Debye model is represented by a semi-circle in the complex plane in Fig. 3. The Cole-Cole plot is located inside the semi-circle defined by Eq. (5). The Davidson-Cole empirical formula (11) is represented by asymmetric contour of impedance. As *l* decreases, the shape of the arc becomes progressively more asymmetric. Finally the Havriliak-Negami connects the properties of Cole-Cole and Davidson-Cole model and enables much more flexibility in the shape of the Nyquist plot presented in Fig. 3.

## Experimental

### Materials

#### New materials PAN/PANI (Fabrication of materials)

In this work we present the formation of doped PANI fibres PAN and their characteristics for the construction of sensory fabrics that dynamically interact with changing environments and respond to the change in controllable manners. For the fibre preparation a polymer of poly(acrylonitrile) (PAN, Good Fellow, UK) was used. It comprised 99.5% (w/w) acrylonitrile and 0.5% (w/w) methyl acrylate. The density of PAN in dimethylformamide (DMF, HSH Chemie, Poland) at 20 °C equaled 2 dl/g.

The PAN/PANI fibers were obtained in three different ways:

By adding powdered polyaniline to a fiber-forming polyacrylonitrile solution. For the fibre preparation a 13.5% (w/w) solution of PAN in DMF was used and the concentration of dopant was set to 1% or 5% (w/w of PAN). First, PANI was added to cold DMF and mixed rubbing for 0.5 h until it was dissolved and grated. Next, PAN polymer was added and the mixture was stirred for approximately 4 h at 40 °C in a tightly covered flask. Afterwards, the mixture was deaerated and cooled down to a room temperature. In this way, the spinning solution was ready for doped PAN fibre formation^29^. After the formation of the fibres with 1% of PANI they were soaked in HCl for 20 hours.

The PAN fibre with polyaniline was prepared by special methods *in situ* synthesis of polyaniline in a spinning solution^29,30^. The fibres were formed with the 13.5% (w/w) of PAN polymer concentration. The presented method of *in situ* synthesis of polyaniline during the preparation of a spinning solution for forming PAN/PANI composite fibers by wet spinning is a new method.

The third way to prepare the PAN fibre with PANI was by easy soaking of formed PAN fibres in a 1% aqueous solution of polyaniline for 20 hours. The fibres were not stretched during their passage through water vapour. The surface of the fiber was porous, and in its interior there were numerous channels (which are numerous pores and cracks). The fibres were formed with the 13.5% (w/w) of PAN polymer concentration.

**Figure 4:**
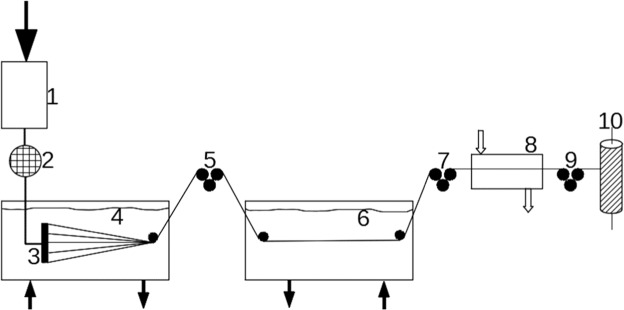
A schematic diagram of the process of doped and nondoped polyaniline polyacrylonitrile fibre preparation: 1- tank with spinning solution, 2- dosing pump, 3- spinneret, 4- solidifying bath (coagulation), 5,7,9 – feeding-receiving points, 6- plasticizing bath, 8- evaporator, 10- multifilament bobbin.

Its rheological properties were assessed with the aid of a rheoviscometer (AntonPaar, Reolab QC). The following parameters were characterized: n – a rheological factor called the flowing factor (where for n = 1 a solution is a Newtonian liquid, for n < 1 liquid the viscosity decreases with the rate of shear strain: shear thinning liquid, while for n > 1 liquid the viscosity increases with the rate of shear strain: shear thickening liquid); k – a consistency factor that denotes the value of apparent dynamic viscosity at a shear rate of 11/s. For the PAN solution without PANI: n = 0.85 and k = 32.85, whereas for PAN with 1% of PANI: n = 0.79 and k = 47.12; for PAN with 5% of PANI: n = 0.70 and k = 53.65; for PAN with PANI *in situ*: n = 0.82 and k = 24.03. The method of fibre preparation was as presented in Fig. 4.

During the fibre pulling process forming nozzles with 500 holes (a spinneret) were used. Each hole was 0.08 mm in diameter. The flow rate through a spinneret channel equalled v = 1.94 m/min. The solidification of the fibres was performed in 60% DMF at 20 °C. Afterwards, plasticization took place in 50% DMF at 40 °C. Finally, finishing was performed in steam at 135–140 °C. Rinsing of the fibres created was performed continuously in H_2_O in a slanting shaft rinser. The fibres were finally dried on bobbins at room temperature (~23 °C). It should be noted that two kinds of fibres were formed. One was lustreless and the other glossy. The glossy fibres were formed as described above, whereas lustreless fibres were formed if the stabilization step in steam was omitted.

### Methods

#### Fibres morphology analysis

A TESCAN VEGA3–Easy Probe (TESCAN Brno, s.r.o., Czech Republic) scanning electron microscope equipped with VEGATG software was used for morphological analysis of the doped polyacrylonitrile fibres (high vacuum mode (SE); accelerating voltage 7–20 kV). Samples both with and without dopants were analyzed. Beforehand, the samples were sputtered with Au (Cressington Sputter Coater 108 auto, UK) for 60 s, resulting in the formation of a 20 nm thick Au layer on the fibres.

#### Mechanical studies

A PC controlled low-load machine was used for testing the mechanical properties of the fibres formed (Zwick Z2.5/TN1S,Germany) according to the PN-EN ISO 2062:2010 standard^31^. The threads were measured in order to examine changes occurring in the structure of the glossy and lustreless threads.

#### Electrical impedance spectroscopy

An ATLAS 0441 High Impedance Analyser (ATLAS-SOLLICH Rębiechowo near Gdańsk, Poland) with data acquisition software was used for impedance spectrum measurements. The impedance measurement range is from 1 Ω to 100 G Ω for wide frequency range from 10 µHz to 1 MHz. The measurements were presented in the form of Nyquist plots. The measurement stand was equipped with specially designed sample holder.

**Figure 5:**
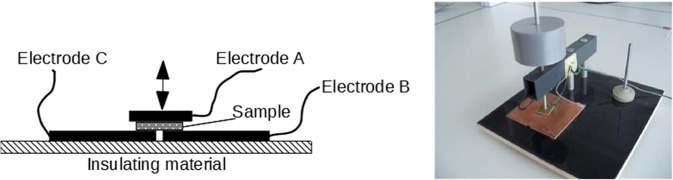
Schematic diagram and real view of sample holder.

As it can be seen in Fig. 5 the sample holder consists of three copper electrodes: A, B and C. The A electrode is a movable one and enables the compressing of a measured sample with a programmed load. The A electrode was a 150 × 150 mm square, while the B electrode was 500 × 500 mm, respectively. The thickness of the measured samples was always much smaller than the linear dimensions of the electrodes and was less than 1 mm, so it was not necessary to provide the stand with a guard ring. Despite of this, a metal shield of the stand was introduced as a protection against the influence of the external EM field. The sample holder construction enables two types of impedance measurements: crossover impedance (between electrode A and connected B and C) and surface impedance (between electrode B and C). Mainly impedance across was used in the measurements, however, the possibility of measuring surface impedance could be interesting due to the potential application of PEM shielding by the tested sample.

Matlab environment was used to prepare fitting program of acquired data into the Debye and non-Debye model curves.

## Results and Discussion

**Figure 6:**
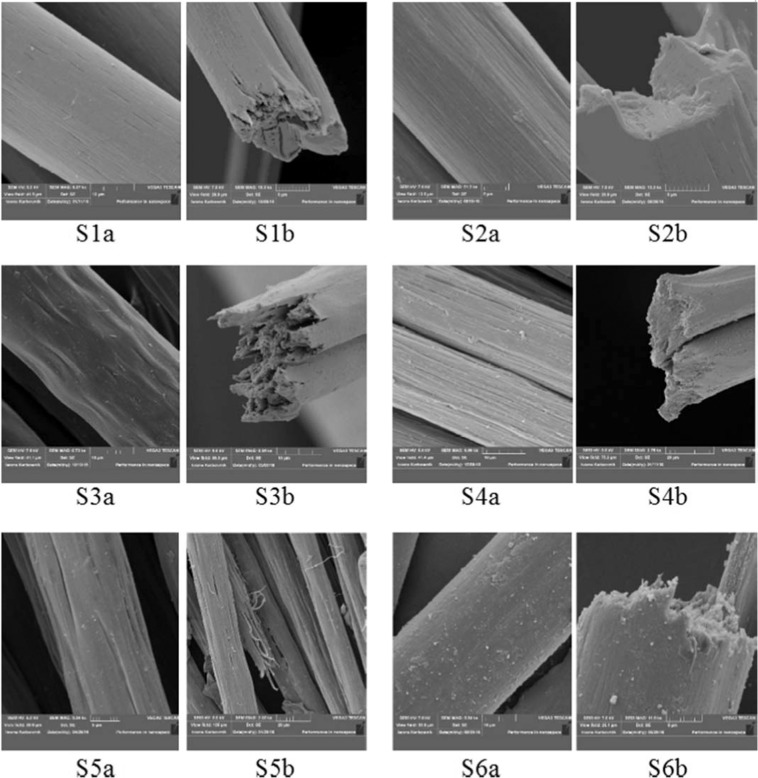
The microstructure of the surface (**a**) and interior (**b**) of the fiber samples from S1 to S6.

During the completed research works, samples from S1 to S6 were obtained (Table 1). All the samples were fibrous systems of different type, composed of two polymers (polyacrylonitrile and polyaniline) with a different length of polymer chains^29^. In addition, the S1 and S2 samples were subjected to an additional treatment involving soaking in hydrochloric acid (HCl). Polyaniline is characterized by a simple and reversible doping with acid chemical reaction, which enables the regulation of its properties, especially connected with electrical conductivity. The electrically conductive form of polyaniline is known as emeraldine in such an oxidation state that when doping with acid the protonation of imino nitrogen atoms in the polymer backbone occurs and charge carriers are induced. In addition, the structure of these fibers (S1 and S2) that can be seen in the SEM picture (Fig. 6) is free of any discontinuities in the form of delamination or cracks. The reason for such differences in the microstructure of fibers may be primarily the difference in the size of the particles that make up both polymers and the amount of fiber stretch obtained in the process of their formation. In addition, as shown in the results of the study^29^, during the synthesis of PANI in the DMF and PAN solution, new interactions between the functional groups of both polymers appeared, which could weaken the interactions between the polyacrylonitrile chains themselves.

**Table 1:** The description of obtained samples with their mechanical properties.

Sample indication	Sample description	Mechanical properties	
		Linear mass, tex	Specific strength, cN/tex
S1 glossy	1% PANI (powder) addition, after forming, the fibers were soaked in an aqueous solution of 3.5% HCl for 20 hours	72.30	23.64
S2 glossy	1% PANI (powder) addition, after forming, the fibres were soaked in an aqueous solution of 35% HCL for 20 hours	75.60	21.67
S3 lustreless	PAN fibers, unstretched in steam, soaked for 20 hours in 1% aqueous solution of PANI	192.97	11.23
S4 glossy	5% PANI (powder) addition, after forming, the fibers were not soaked in HCl	88.97	19.56
S5 glossy	PANI *in situ*	71.47	25.07
S6 lustreless	PANI *in situ*, the fibers not subjected to stretching in steam	100.03	18.39

The samples S5 and S6 were composed of PAN fibres with PANI by *in situ* reaction. The worst conductive properties (the best dielectric properties) were obtained for S6 sample. In comparison to S5, S6 was not subjected to stretching in steam, which results in many pores and cracks in the fibres interior. During the *in situ* synthesis in the spinning solution, new interactions between functional groups of both polymers appeared which could weaken interactions between the polyacrylonitrile chains themselves^29^. These interactions made the fibers easier to delaminate than to break during stretching. The fiber structure of samples S5 and S6 is discontinuous and contains many defects of different type. It is definitely more heterogeneous as opposed to fiber samples S1, S2 and S4.

The microstructure of the surface and interior of the fibers were observed using a scanning electron microscope. Photographs of fiber samples are provided in the Fig. 6. It was found that both the surface morphology and the inside of the fibers are different for individual samples. Surface of fibers doped with polyaniline powder in an amount of 1% by weight in relation to the polyacrylonitrile used, treated with hydrochloric acid, is smooth (samples S1 and S2). On the surface of S4 sample, deep furrows are clearly visible. The fibers contained polyaniline powder in an amount of 5% by weight and they have not been treated with hydrochloric acid.

Cross-sections of polyacrylonitrile fibers, unstretched in steam and soaked in a 1% solution of polyaniline (sample S3), show numerous channels (pores and cracks). The PAN/ PANI fibers *in situ* (sample S5) were delaminated and broked during the spinning process at the stage of stretching them in steam, and therefore this step was omitted and this way the S6 fiber sample was obtained. The structure of PAN/ PANI fibers *in situ* (samples S5 - subjected to stretching in steam and S6 - not subjected to stretching in steam) is discontinuous and is definitely more inhomogeneous, as opposed to samples of fibers doped with polyaniline powder (S1, S2 and S4).

Polyaniline is an intensely tested material mainly due to its high electrical conductivity, low price and an easy method of obtaining. However, the biggest difficulty associated with its use is its low mechanical strength and processing problems. For this reason, the fibers were made from a mixture of polyaniline with fiber-forming polyacrylonitrile, which in this system is a polymer responsible for mechanical strength.

The fiber linear mass was determined using the Eq. (15):

15 $${m}_{l}=\frac{m}{l}\cdot 1000\,[\frac{g}{m}\cdot 1000=tex]$$

where:*m* - mean sample mass, *l* - sample length.

The linear masses of the unstrached in steam fibers (S3, S6) were visibly higher than the samples of fibers subjected to this stretch (S1, S2, S4, S5), which was reflected in the specific strength of fiber multifilament values. Stretched samples were even twice as strong as those without stretching in steam (Table 1).

Good mechanical parameters of S1, S2 samples were also influenced by the presence of low-molecular PANI fractions (1 mass %) in the fiber structure^29^. The low molecular PANI mixed well with pure polyacrylonitrile therefore the obtained fibers have good mechanical properties (Table 1) and a smooth structure (Fig. 6). In addition, polyaniline can occur in three oxidation states, of which only the emeraldine salt has electrical conductivity at the semiconductor level, i.e. about 1 S/cm. Emeraldine salt is obtained as a result of modifications to emeraldine base, among others such a compound as HCl. Each of the PANI forms can be transformed in a reversible oxidation or reduction reaction process, during which there is a change in electrical conductivity^29^.

The analysis of the above leads to the conclusion that a larger amount of polyaniline (5 mass %) (S4, Table 1) introduced into the fibers in order to do the desired increase in conductivity, perfectly mixed with polyacrylonitrile and formed a homogeneous structure. However, this addition has already resulted in a less smooth fiber structure, which was reflected in the reduction of S4 fiber strength.

During the stretching of fiber-forming polymers, their crystalline structure is shaped, which resulted in the increase of the mechanical strength of the fibers. However, as it has already been mentioned, the fiber samples S3 and S6 were not subjected to stretching in steam. The low mechanical strength of these fibers was associated primarily with the lack of stretching and with their heterogeneous structure and low degree of crystallinity^29^.

**Figure 7:**
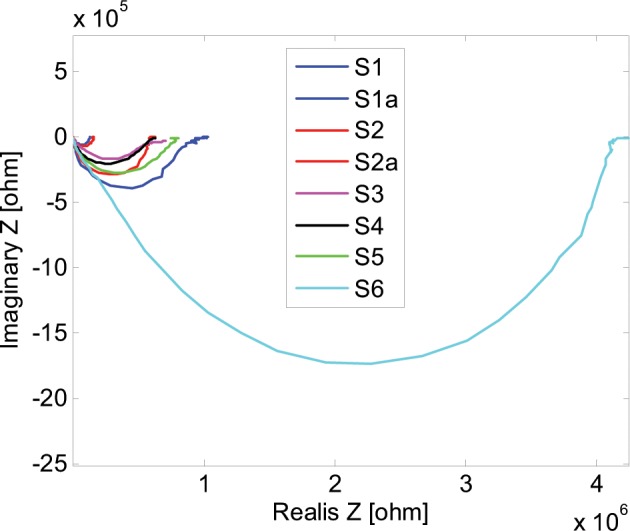
Nyquist plots of impedance spectra of samples listed in Table 1.

In Fig. 7 Nyquist plots of cross (S1, S2, S3, S4, S5, S6) and surface (S1a, S2a) impedance spectra for all the samples that were measured were presented. The impedance spectra was obtained using the impedance spectroscopy method in the frequency range from 0.01 Hz to 100 kHz. The impedance spectra shows clearly that the resulting complex impedance is dependent on the process parameters that were subjected the acquired samples of fibres. With the increasing sample number (from S1 to S6) the Nyquist plots of cross impedance expand, maintaining almost the same curve shape. Samples S1a and S2a that were obtained for surface measurements also confirm this tendency but for different impedance values. From the presented Nyquist plot also the quasi DC resistance parameter of the sample can be read. It can be estimated for the value that the Im(Z) equals zero. The lowest impedance/resistance value is achieved for the S1 sample. The DC resistance is in the range of 120 kΩ. Sample S1 shows the most conductive properties of all the samples which is the result of 1% of PANI addition. After formation the sample was soaked in 3.5% HCL aqueous solution. Sample S2 can be characterized by almost the same electrical and mechanical properties as sample S1. The DC resistance is in the range of 150 kΩ. Samples S3 and S4 present very similar properties to the Nyquist plot point of view presented in Fig. 7. It may be a little confusing because the fibres samples were prepared in a completely different way. Sample S3 was composed of unstretched in steam PAN fibres that, after the formation process, were soaked for 20 hours in 1% aqueous solution of PANI conductive polymer. In contrary to sample S4 which was composed of PAN fibres with 5% of PANI powder addition, without soaking in HCl. This case shows clearly that similar impedance curves can be obtained for post formation processing of PAN fibres – S3 as well as for polymeric additions to the formatting mass – S4. In both cases (S3, S4) the DC resistance is in the range of 700 kΩ and similar dependence on frequency. Sample S5 shows slightly worse conductive properties in comparisons to samples S3 and S4. The DC resistance is in the range of about 800 kΩ and the impedance curve maintains the same shape. The surface impedance spectra were made for samples S1 and S2 and the corresponding curves are marked S1a and S2a. As it can be seen in Fig. 7 the surface measurements show worse conductive properties of DC and AC impedance point of view compared to cross impedance measurements of the same samples. S2a sample can be comparable in the curve shape and values to the S3 and S4 samples and S1a is most similar to the S5 sample.

**Figure 8:**
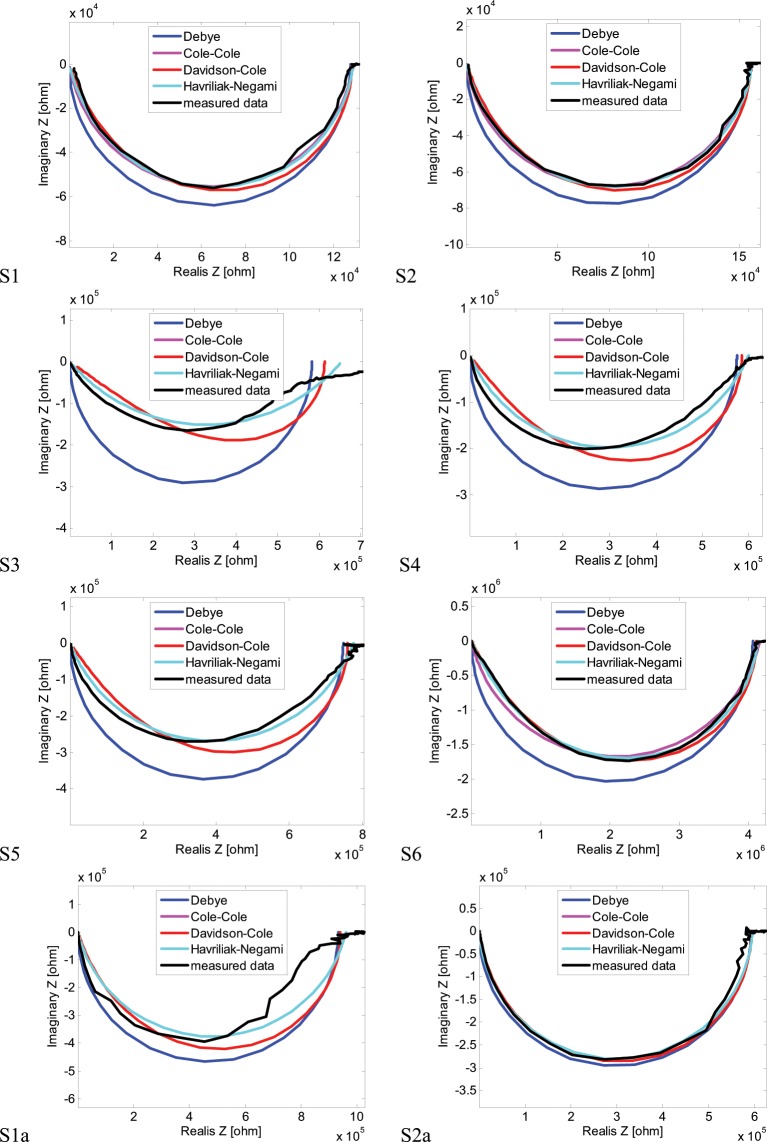
Nyquist plots of impedance spectra for all the samples analyzed compared with best model impedance curves.

The dynamical properties of impedance spectra of the samples presented in Fig. 7 can be analysed using different model approaches presented in chapter 2. Figure 8 shows the impedance spectra for all the samples considered in Table 1 with the model fitting analysis. The four model types were considered: Debye, Cole-Cole, Davidson-Cole and Havriliak-Negami. Using Matlab based optimizing procedure model parameters were changed trying to best fit each model into measurement data. The procedure was based on least-square method minimizing the error between model and measurement curves. Each chart in Fig. 8 comprises different measurement data set (for different sample) and four best fitting model approaches for Debye, Cole-Cole, Davidson-Cole and Havriliak-Negami models respectively. For comparing purposes the fitting error in the form of mean absolute percentage error (MAPE) was defined:

16 $$Error\,Q=\frac{100 \% }{n}\mathop{\sum }\limits_{i=1}^{n}|\frac{{Z}_{\mathrm{mod}}-{Z}_{meas}}{{Z}_{\mathrm{mod}}}|$$

where:

${Z}_{mod}$- best fitted model impedance

${Z}_{meas}$- impedance curve acquired from impedance spectroscopy measurements

*n* – number of frequency points

**Figure 9:**
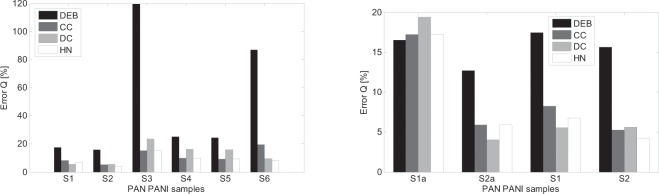
Fitting error for all samples impedance spectra.

Equation (16) enables to compare the quality of fitting process for different models. It was shown, using the bar graphs in Fig. 9.

Analysing the waveforms shown in Fig. 8 in comparison with fitting errors presented in Fig. 9 it can be seen that the best fit is achieved for the so called fractional models of different kind i.e.: Cole-Cole (CC), Davidson-Cole (DC) and Havriliak-Negami (HN). The simplest, integer – Debye (DEB) model shows the biggest difference between data and model curves with one exception – S1a sample. As it can be seen, especially in Fig. 9 the best fit is achieved for a different fractional model depending on the sample considered. For samples S1 and S2a the smallest error is presented for Davidson-Cole model. The best fit for Havriliak-Negami model is achieved for samples S2 and S6. In the case of the samples: S3, S4 and S5 the two models: Cole-Cole and Havriliak-Negami present similar results of the smallest error. It can also be seen in Fig. 7 for S3, S4 and S5 where CC and HN curves overlap.

In Tables 2 and 3 the model parameters summary is presented. It shows all the parameters values, for all Debye and non-Debye models, that were acquired for all the samples during the fitting process trying to minimize the error (16). The model’s parameters in bold show the model that present the smallest fitting errors compared to other models for each sample. It is corresponding to the bar graphs presented in Fig. 9. Analysing the parameters values presented in Tables 2 and 3 only the values shown in bold will be taken into account as these model present the smallest errors of all. However, it is difficult to compare the tendency showing each parameter change as it refers to different model. To enable the comparison the most complex – Havriliak-Negami model will be considered. It is the most complicated model as it covers all the other models (Debye, Cole-Cole, Davidson-Cole) in respect to special values of α and γ parameters. In other words, in Table 2 and Table 3, in case of non-HN models one can see model reduction to special cases like: DEB, CC or DC.

**Table 2:** Model parameter values for different samples.

Sample							
Model		S1	S2	S3	S4	S5	S6
DEB	K	0.127e6	0.155e6	0.582e6	0.574e6	0.747e6	4.067e6
	T	0.154e-4	0.287e-4	1.305e-4	0.322e-4	0.408e-4	5.233e-4
CC	K	0.128e6	0.157e6	**0.663e6**	**0.599e6**	**0.752e6**	4.167e6
	T	0.156e-4	0.290e-4	**2.017e-4**	**0.367e-4**	**0.451e-4**	5.315e-4
	α	0.904	0.908	**0.552**	**0.743**	**0.771**	0.861
DC	K	**0.128e6**	0.156e6	0.612e6	0.585e6	0.759e6	4.116e6
	T	**0.213e-4**	0.392e-4	7.536e-4	0.756e-4	0.876e-4	8.544e-4
	γ	**0.783**	0.791	0.395	0.585	0.612	0.695
HN	K	0.128e6	**0.157e6**	**0.653e6**	**0.599e6**	**0.775e6**	**4.137e6**
	T	0.174e-4	**0.324e-4**	**2.017e-4**	**0.367e-4**	**0.451e-4**	**7.511e-4**
	α	0.928	**0.933**	**0.552**	**0.743**	**0.771**	**0.945**
	γ	0.923	**0.920**	**1.000**	**1.000**	**1.000**	**0.776**

**Table 3:** Model parameter values for samples S1 and S2.

Sample Designator					
Model		S1a	S2a	S1	S2
DEB	K	**0.940e6**	0.594e6	0.127e6	0.155e6
	T	**3.617e-4**	1.917e-4	0.154e-4	0.287e-4
CC	K	0.960e6	0.596e6	0.128e6	0.157e6
	T	2.768e-4	1.739e-4	0.156e-4	0.290e-4
	α	0.845	0.961	0.904	0.908
DC	K	0.940e6	**0.594e6**	**0.128e6**	0.156e6
	T	3.617e-4	**1.917e-4**	**0.213e-4**	0.392e-4
	γ	0.783	**0.922**	**0.783**	0.791
HN	K	0.960e6	0.596e6	0.128e6	**0.157e6**
	T	2.768e-4	1.739e-4	0.174e-4	**0.324e-4**
	α	0.845	0.961	0.928	**0.933**
	γ	1	1	0.923	**0.920**

**Figure 10:**
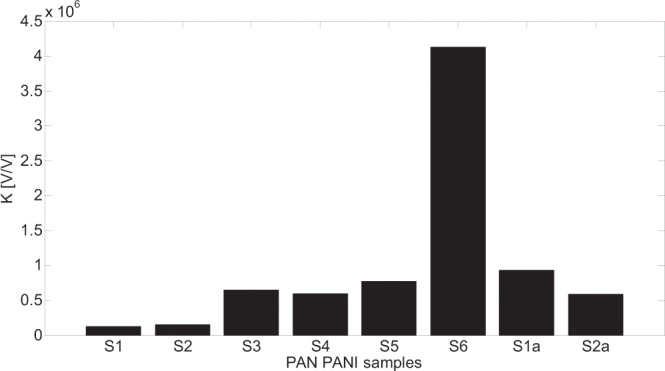
K parameter changes for all the models that returned the smallest fitting error.

**Figure 11:**
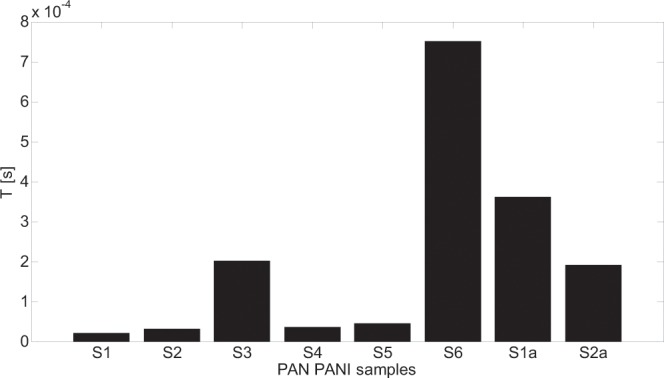
T parameter changes for all the models that returned the smallest fitting error.

**Figure 12:**
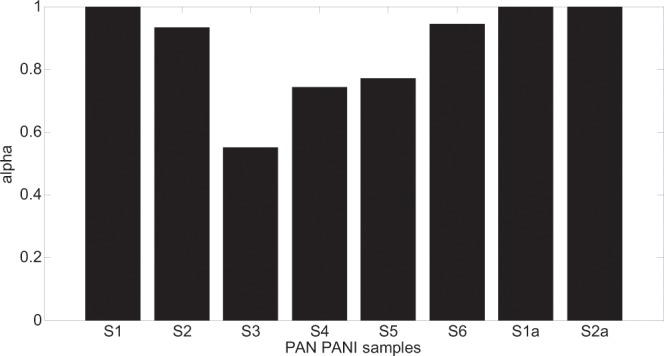
Alpha parameter changes for all the models that returned the smallest fitting error.

**Figure 13:**
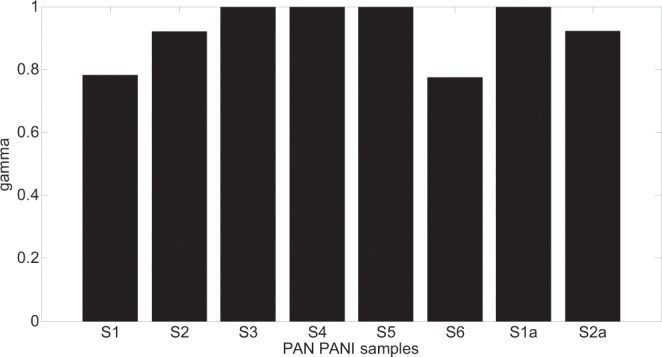
Gamma parameter changes for all the models that returned the smallest fitting error.

The proposed solution is illustrated in four bar graphs that show the parameters changes for all the models that returned the smallest fitting error (in bold in Tables 2 and 3). As it is seen in figures from Figs. 10–13 all parameters values (K, T, alpha, gamma) are presented for all the samples but for the minimum values of fitting error other than in HN, model α or γ equalling 1 were required.

Generally speaking the value of K parameter of the analyzed models is proportional to the complex impedance of samples shown as a radius value (expansion) of Nyquist plots shown in Fig. 7. Comparing the curve shapes presented in Fig. 7 with the K parameter values for the same sample it can be seen that the K parameter value increases with the expansion of the Nyquist plot of the sample. On the other hand the impedance value of the samples reflected by K parameter is dependent on the value and the way of arrangement of PANI conducting polymer. Despite the fact that samples S1 and S2 contain only one weight percent of PANI conducting polymers they are characterized by the smallest impedance value of all the samples, reflected by K parameter. It is caused by uniform distribution of conducting form of PANI polymer in these samples. In contrary, S4 sample, characterized by higher value of K parameter (higher impedance), was composed with the 5% addition of PANI polymer. It was the result of the lack of conducting form of PANI polymer caused by the lack of HCl redopping. In turn the S3 sample (higher K value compared to S4 sample) is characterized by different spatial structure than other samples. The S3 sample contains a number of air spaces between PAN and PANI polymers due to the lack of PAN fiber stretching. The S6 sample is characterized by the maximum value of K parameter. In this case the small molecule PANI conducting polymer^29^ was rinsed out from inside to the outer layer of the fiber, which causes the increase in resulting impedance value. The S5 sample was prepared in the same way as S6 sample but was subjected to stretching in steam that led to a decrease in impedance value.

The changes of T parameter shown in Fig. 11 reflect the changes in relaxation times (time constants) of the samples, that can be connected to capacitive properties of PAN/PANI polymeric system. Minimum values of relaxation times were obtained for the samples that were subjected to all stages of production process (including the stretching in steam) i.e. samples: S1, S2, S4 and S5. Not stretched samples (S3 and S6) are characterized by higher values of relaxation times compared to S1, S2, S4 and S5. In the case of S3 sample it is the effect of existence of a large number of airspaces in the volume of the fibres. In the case of S6 sample it is caused by the fact that small molecule PANI conducting polymer was rinsed out from inside to the outer layer of the fiber increasing dielectric properties.

The changes of alpha and gamma parameters, shown in Figs 12 and 13 in respect to Eqs. (7,12) and (14) show that not every sample can be described using ordinary differential equations. The cases, presented in Figs. 12 and 13, for which the alpha and gamma parameters values differ from 1 show the need to use of fractional differential equations that have their consequences in CC, DC and HN models. (Only the DEB model is based on ordinary differential equations). Analysing bar graphs in Figs. 12 and 13 it can be seen that almost every sample has at least one parameter (alpha or gamma) that differs from one. Based on the information from Tables 2 and 3 it can also be concluded that especially for HN model there is a strict relation between alpha and gamma parameters i.e. alpha > gamma. The cases for which gamma > alpha are the ones for which gamma = 1 and the HN model reduces to CC model. Another consequence of introducing the fractional order models is the modification of capacitive elements leading to constant phase elements (CPE) – Eq. (9). The minimum of fitting error presented in Fig. 9 leading to good compatibility between the measured and model impedance curves for fractional models reveals that the obtained resulting polymeric structure is very complicated and inhomogeneous. It can be compared to complicated process of modelling of the electrochemical double layer capacitor (EDLC)^32^.

## Conclusions

During the completed research works, complex fibrous systems with dielectric properties were obtained. Such dielectric systems show significant deviations from the simple Debye model, which was influenced by several factors, that include:

- the length of macromolecules (particles) of polymers included in the system
- the distribution of polyaniline particles in the polyacrylonitrile matrix
- the presence of a strong reducer in the form of HCl in the PAN / PANI system
- occurrence of defects (cracks, pores) inside these systems and discontinuities on the surface of fibers, resulting from the assumed spinning conditions.

The above factors have influenced the fact that the samples impedance can be better described by the fractional CC, DC, HN models and is the result of the CPE (constant phase elements) occurrence. In most of the cases, the smallest fitting error was found for the CC and HN models. The CC model is a typical fractional order model that describes the structure of the so-called constant phase element. This model fits into the experimental impedance results with the smallest error for the samples S3, S4 and S5. In contrast, the HN model allows the most variations in the parameter space and fits well the impedance of the S2, S6 samples, while the description of impedance samples: S3, S4, S5, despite the admission of the variable parameter γ, have been reduced to the CC model as γ = 1.

The presence of fractional models in the description of impedance spectra is caused by the occurrence of a complex relaxation process in the samples. A simple, Debye relaxation model (with only one relaxation time) can only be applied to the description of the S1a sample.

An attempt was made to correlate the physico-chemical properties of the tested samples with the parameters of the models for which the smallest matching error between modeled and experimental impedance spectra was obtained. The K parameter is responsible for the gain value (impedance module) and shows that its minimum value has been obtained for the S1 and S2 samples and the maximum for S6 sample. This can be related to the distribution of conductive particles in the polymer matrix.

The value of the T coefficient corresponds to the time of inertia (relaxation time). When α and γ equal 1, the case of a simple relaxation model occurs that is only valid for sample S1a. In other cases, complex relaxation time models can be observed, which are characterized by α or γ different from 1. The most complex cases are those for which both α and γ are smaller than 1, e.g. samples: S2 and S6. This is the result of the following factors:

- the porosity of the fiber structure – samples: S3, S4, S5 and S6
- the local distribution of conduction paths (lack of uniformity of samples) – samples: S2, S3, S4 and S5
- the statistical distribution of grain resistances in a sample (particle size distribution and relaxation times).

All these factors influenced the fitting process of the shape and values of individual models to the experimentally acquired impedance spectra.
